# An Unexpected and Unpredictable Emergence of Muscle-Invasive Bladder Cancer in a Patient Successfully Treated With Nivolumab for Small-Cell Bladder Cancer

**DOI:** 10.7759/cureus.32244

**Published:** 2022-12-06

**Authors:** Thiago Guimarães, Hugo Pinheiro, João Pimentel, Hermínia Pereira, Luís Campos Pinheiro

**Affiliations:** 1 Urology, Centro Hospitalar Universitário de Lisboa Central, Lisbon, PRT; 2 Anatomic Pathology, Centro Hospitalar Universitário de Lisboa Central, Lisbon, PRT

**Keywords:** small-cell bladder cancer, urothelial bladder cancer, follow-up, nivolumab, immune checkpoint inhibitors

## Abstract

Small-cell bladder cancer (SCBC) is a rare subtype of bladder cancer with aggressive behavior and poor prognosis. Here, we report the case of a 50-year-old man who presented with hematuria for one month. A computed tomography scan showed an exophytic lesion on the right posterolateral wall of the bladder and a single liver metastasis with a 14 mm diameter. Transurethral resection of the bladder tumor was performed, and postoperative examination of the specimen showed muscle-invasive SCBC. Initially, the patient was treated with neoadjuvant chemotherapy. Rapid clinical and imaging deterioration was observed after the premature end of cisplatin and etoposide therapy. Second-line therapy with nivolumab demonstrated systemic and local complete response. However, the patient was further diagnosed with unpredictable and unexpected urothelial muscle-invasive bladder cancer. After 76 months of regular follow-up, imaging workup did not demonstrate SCBC recurrence or urothelial bladder cancer progression. This report highlights this disease’s rarity and severity and no typical or even pathognomonic clinical and radiological presentation. Therefore, histopathology and immunohistochemistry findings play a key role in diagnosis. Immunotherapy has opened a new window in cancer treatment and maybe SCBC patients can benefit from it.

## Introduction

Bladder cancer shows extensive genetic and pathological heterogeneity. The urothelial subtype accounts for more than 90% of bladder cancer cases [1]. However, histologic variants may be present in their pure form or more commonly associated with the urothelial subtype. Abrahams et al. demonstrated that mixtures of small-cell bladder cancer (SCBC) with urothelial cancer were present in 70% of bladder cancer specimens [2]. Divergent differentiation and pure histologic variants of bladder cancer were highlighted in the 2016 World Health Organization classification [3]. They have been associated with poor outcomes after treatment. SCBC represents 0.7% of all primary bladder malignancies [4]. The etiology and pathologic mechanism triggering the onset of SCBC are poorly understood. Tobacco smoking is the main risk factor for bladder cancer including the SCBC subtype [4]. The cases reported in the literature suggest that these tumors most frequently affect adult males. The average age of onset is 67 years [2]. In most cases, SCBC is asymptomatic in its earlier stage. However, relevant complaints occur based on tumor growth and its location. The most common but unspecific symptom is gross hematuria [2].

The identification of bladder cancer variants and subtypes is important for treatment decisions and outcomes [3]. Based on the European Association of Urology and European Society for Medical Oncology (EAU/ESMO) consensus, neoadjuvant chemotherapy can play a role in the treatment of patients with mixed urothelial and SCBC and responders may benefit from the management of local excision [5]. The standard treatment for patients with muscle-invasive urothelial bladder cancer with or without variants is radical cystectomy. However, randomized data on the treatment of patients with pure SCBC are lacking and limited [4]. SCBC shares biological similarities with small-cell lung cancer (SCLC). Therefore, the treatment strategy is usually related to those proposed for SCLC [6]. Patients with SCBC receiving either monotherapy or combination therapy have an estimated median survival of 12 to 24 months. Without treatment, the prognosis is bleak with an estimated survival time of four or five months [7]. Recent efforts have focused to determine the benefit of programmed death protein 1 (PD-1)/programmed cell death ligand 1 (PD-L1) checkpoint inhibitors in the treatment of metastatic bladder cancer and SCLC. Based on this, recent reports have suggested the potential role of immune checkpoint inhibitors in the treatment of SCBC.

## Case presentation

**Figure 1:**
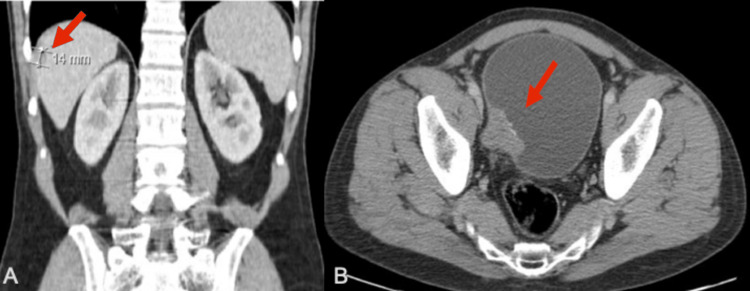
Imaging findings at presentation. A: CT scan showing a single liver metastasis (red arrow). B: CT scan showing an exophytic lesion on the right posterolateral wall of the bladder (red arrow). CT = computed tomography

In July 2017, a 50-year-old male patient presented to our urological clinic with one-month-long gross hematuria. His relevant medical history included arterial hypertension and tobacco smoking. A computed tomography (CT) scan showed an exophytic lesion on the right posterolateral wall of the bladder involving the homolateral ureteral orifice. A single liver metastasis with a 14 mm diameter was also detected in segments VI/VII (Figure 1).

**Figure 2:**
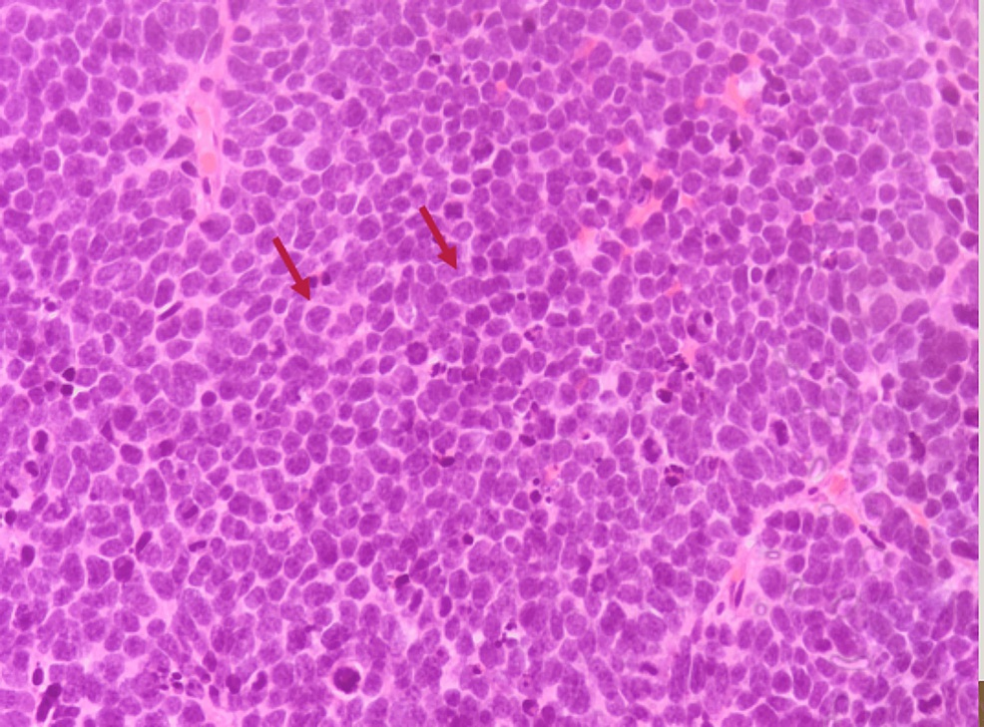
Histopathological findings of small-cell bladder carcinoma in our patient. Hematoxylin and eosin stain image (40× magnification) showing malignant cells with large nuclei and scant cytoplasm (red arrows).

**Figure 3:**
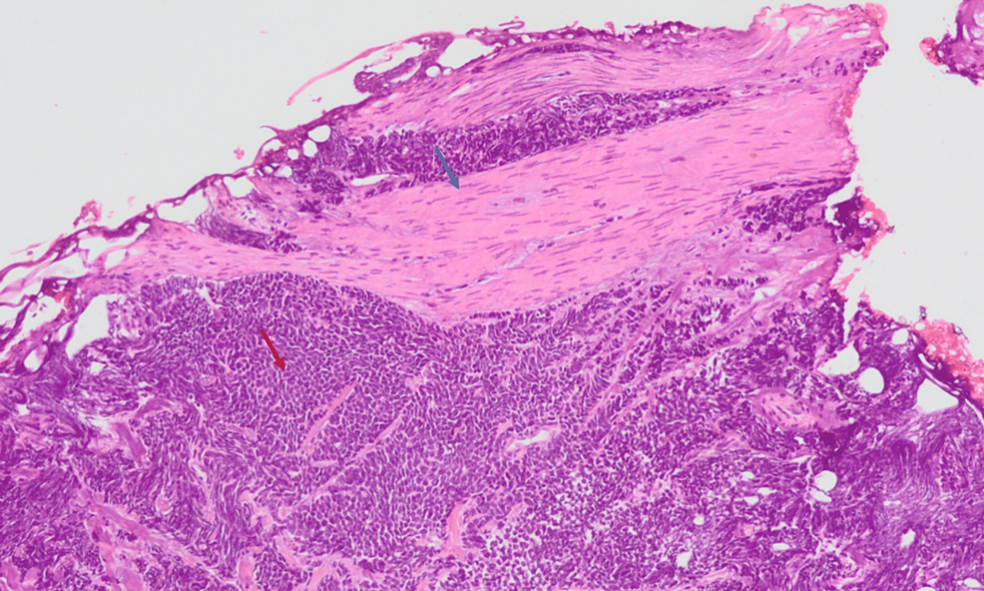
Muscle-invasive small-cell bladder cancer. Hematoxylin and eosin stain image (10× magnification) showing sheets and nests of small cells with hyperchromatic nuclei and scant cytoplasm (red arrow) with increased mitotic index invading the muscle layer of the bladder (blue arrow).

**Figure 4:**
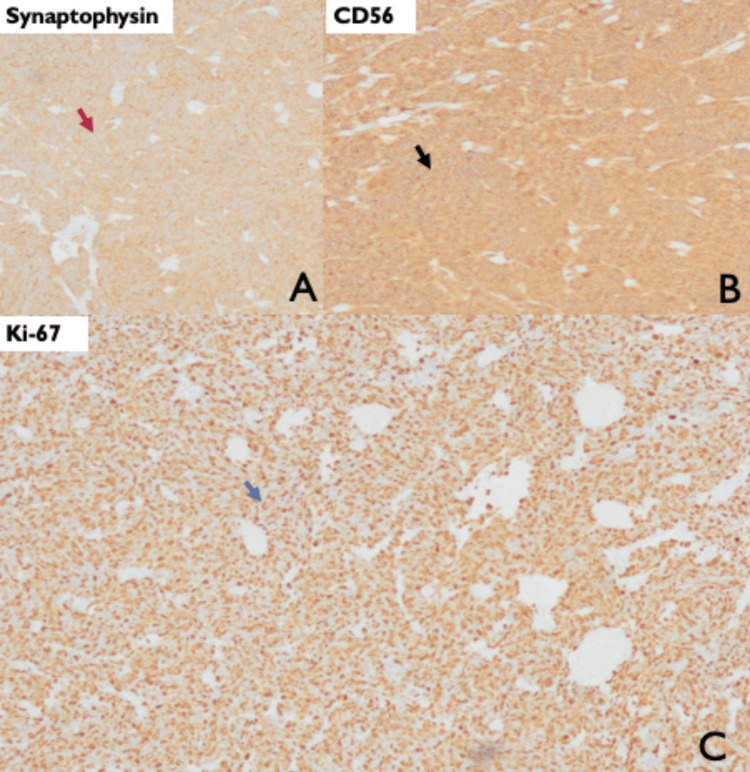
Neuroendocrine markers and Ki-67 expression. Immunohistochemistry analysis of bladder tissue (10× magnification). A: Synaptophysin expression is identified by the brown staining in the cytoplasm of tumor cells (red arrow). In this case, synaptophysin shows homogenous strong positivity. B: CD56 stain with brown color highlighting positive neuroendocrine tumor cells (black arrow). C: Immunopositivity for Ki-67 is defined as brown staining in the tumor nucleus (blue arrow). In this case, the expression of Ki-67 was almost 100%.

Bone scintigraphy and cranial magnetic resonance imaging (MRI) were negative for bone and brain metastases. The patient was proposed transurethral resection of the bladder tumor (TURBT). Histological examination revealed malignant cells with large nuclei and scant cytoplasm arranged in sheets (Figures 2, 3). Immunohistochemical stains showed high positivity for neuroendocrine markers (synaptophysin and CD56) and cell proliferation (Ki-67), as demonstrated in Figure 4. Based on these findings, an accurate diagnosis of pure muscle-invasive SCBC was made.

**Figure 5:**
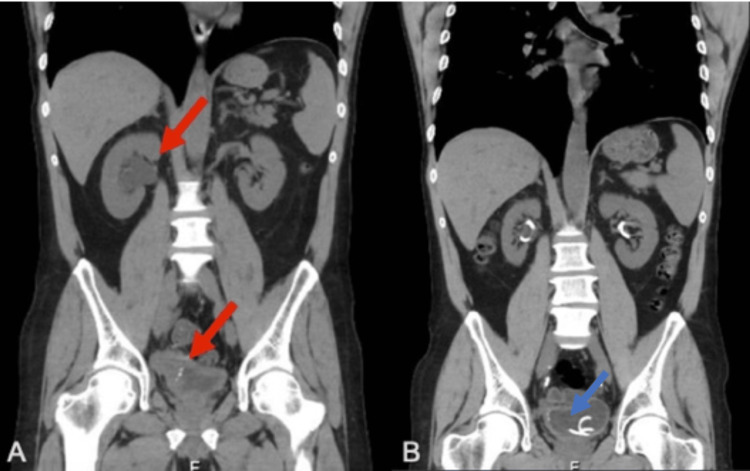
Imaging findings before and during the treatment with nivolumab. A: CT scan showing a large bladder tumor and right hydronephrosis and hydroureter resulting from the extrinsic compression of the right ureter by metastatic pelvic lymph nodes and tumor infiltration of the right ureteral orifice (red arrows). B: Complete bladder response during the treatment with nivolumab (blue arrow). CT = computed tomography

After a multidisciplinary evaluation, the patient was accepted for six cycles of cisplatin 75 mg/m^2^ and etoposide 100 mg/m^2^. Imaging evaluation during chemotherapy showed a partial response of bladder tumor and liver metastasis. However, the patient developed nephrotoxicity and neutropenia. After the premature interruption of chemotherapy, rapid clinical and imaging deterioration was observed. In addition to multiple liver metastases, a follow-up CT scan demonstrated a large bladder tumor and right hydronephrosis and hydroureter resulting from the extrinsic compression of the right ureter by metastatic pelvic lymph nodes and tumor infiltration of the right ureteral orifice (Figure 5A). A bilateral double J stent was inserted at that time to restore kidney function. In May 2018, the patient initiated off-label immunotherapy with nivolumab 240 mg intravenously administered twice a week for 31 months. The patient achieved a complete response concerning liver metastases, locoregional lymph nodes, and bladder tumor (Figure 5B).

**Figure 6:**
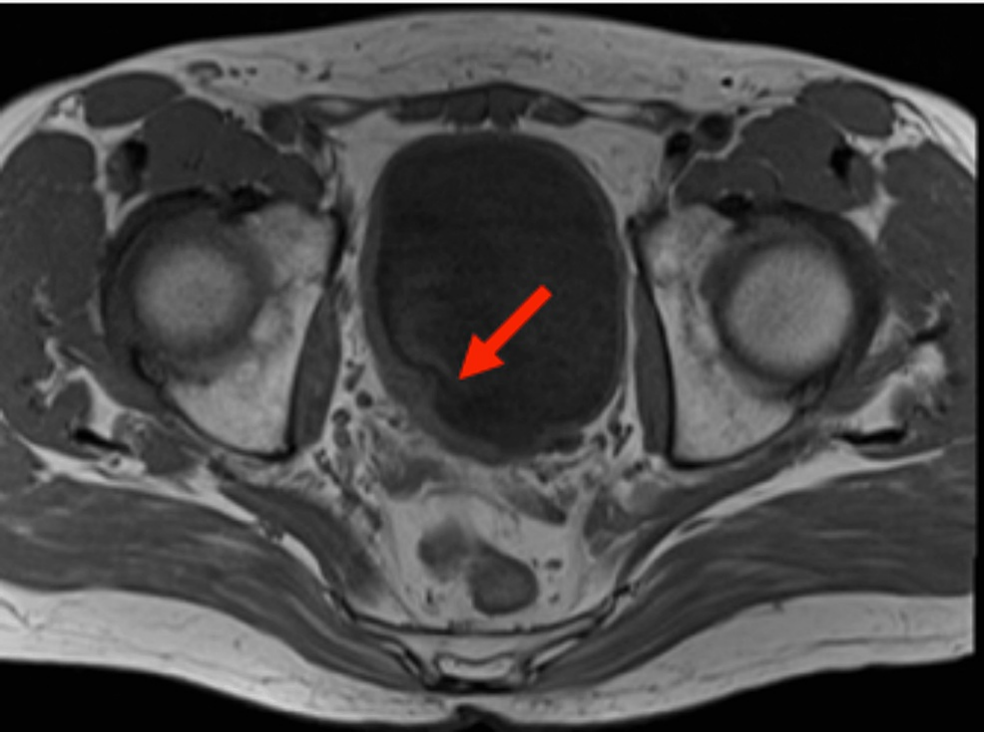
Pelvic MRI findings after the discontinuation of nivolumab. Pelvic MRI showing an irregular thickening of the right-sided wall of the bladder (red arrow). MRI = magnetic resonance imaging

During immunotherapy, an intensive follow-up protocol was initiated. Chest-abdominal-pelvic CT scan, cystoscopy, and urine cytology were performed every three months. Physical examination and laboratory tests, including hemogram, renal function, and liver function, were also routinely performed. Treatment with nivolumab was well tolerated. However, the patient initially complained of transitory arthralgia and skin rash. In December 2020, a follow-up cystoscopy showed relapse in the area of previous resection and trigonous. Nivolumab treatment was suspended and he was submitted to TURBT. Histopathological examination demonstrated a different histological pattern. There was no evidence of small-cell cancer. However, the presence of muscle-invasive urothelial bladder cancer was described. Control positron emission tomography using fluorodeoxyglucose (PET-FDG) revealed increased uptake in the posterior median region of the vesical wall. Because this was an exceptional case of survival, the patient was proposed consolidating local treatment with radical cystectomy or external radiotherapy but he refused both options, claiming a possible decline in his quality of life. A positive recurrence of the bladder tumor was observed cystoscopically. The pelvic MRI demonstrated an irregular thickening of the right-sided wall of the bladder and the absence of suspicious liver lesions or suspected lymph nodes (Figure 6).

In June 2022, TURBT was carried out and the pathology report showed T1 high-grade urothelial bladder cancer. The patient remains in regular follow-up after 76 months of the initial diagnosis of SCBC without systemic or local progression of urothelial bladder cancer. Currently, the patient is proposed for BCG intravesical therapy.

## Discussion

Inhibition of the PD-1/PD-L1 checkpoint has demonstrated significant advantages in patients with advanced and metastatic urothelial and non-urothelial bladder cancer [5]. In 2016, pembrolizumab induced a complete response in a patient with metastatic and chemorefractory bladder cancer with small-cell variant histology [8]. This report sparked interest among researchers in using immunotherapy to treat SCBC. Durvalumab and tremelimumab were evaluated in metastatic, non-urothelial cancer of the genitourinary tract in a phase II trial [9]. Seven patients with pure or mixed urothelial/small-cell cancer were included. However, this trial failed to demonstrate efficacy. Finally, Hoffman-Censits et al. reported the case of a 52-year-old male patient who was treated with four cycles of etoposide and cisplatin chemotherapy before radical cystectomy. After 23.5 months of surgery, the patient developed bone metastases. After second-line chemotherapy, the patient initiated nivolumab therapy for 39 months. The patient achieved a complete and durable response of 82.9 months [10].

Nevertheless, it is important to highlight that SCBC is histologically similar to SCLC. Despite the remarkable sensitivity to initial chemotherapy, the majority of patients with SCLC and SCBC develop resistant/refractory chemotherapy disease. Atezolizumab and durvalumab have a role as first-line therapy for advanced SCLC along with etoposide and platinum [11]. In 2018, the Food and Drug Administration transiently approved nivolumab for SCLC after treatment failure of platinum-based chemotherapy [12]. This also supported our choice for the use of nivolumab in our patient.

Nivolumab is a human monoclonal immunoglobulin G4 antibody that blocks PD-1. Nivolumab as monotherapy has proven to be safe and efficient in multiple solid tumors, including in recurrent metastatic urothelial carcinoma [13].

We believe that our patient represents an interesting case of the clinical efficacy and safety of nivolumab in metastatic SCBC. Although the relapse of muscle-invasive urothelial bladder cancer in a patient under nivolumab treatment was unpredictable and unexpected, it is necessary to answer the question of how a patient develops a pure muscle-invasive urothelial bladder cancer during immunotherapy treatment. Muscle-invasive urothelial bladder cancer onset during the treatment with nivolumab can be partially explained by resistance mechanisms to immune checkpoint inhibitors of undiagnosed cluster of urothelial bladder cancer cells, and persistence of risk factors exposure as tobacco smoking [2,14-16]. However, the mechanisms of acquired resistance to checkpoint inhibitors are not completely understood [17].

Checkpoint inhibitors are increasingly evaluated in the neoadjuvant setting of muscle-invasive urothelial bladder cancer with promising results and can be possibly extended for those with non-urothelial variants in the future [18]. However, it is not yet approved in this setting. We hope that our case report motivates researchers to fill the gap in prospective trials evaluating the use of different treatment regimens specifically designed for patients with SCBC.

## Conclusions

SCBC is an aggressive malignancy. The diagnosis is usually performed at advanced stages. Immunohistochemistry plays an important role in the diagnosis of SCBC. The strategy for the treatment of SCBC is derived from the treatment of its counterpart SCLC and urothelial bladder cancer. Further investigations are needed to improve our knowledge of the pathophysiology, diagnosis, and treatment of this rare disease. Although immune checkpoint inhibitors showed promising therapeutic outcomes in SCBC and metastatic urothelial bladder cancer, more efforts are needed to understand the mechanisms of tumor immune resistance.
